# Food Waste in Schools: A Pre-/Post-test Study Design Examining the Impact of a Food Service Training Intervention to Reduce Food Waste

**DOI:** 10.3390/ijerph18126389

**Published:** 2021-06-12

**Authors:** Sara A. Elnakib, Virginia Quick, Mariel Mendez, Shauna Downs, Olivia A. Wackowski, Mark G. Robson

**Affiliations:** 1 Department of Family and Community Health Sciences, Rutgers University, New Brunswick, NJ 08901, USA; marielrodz14@gmail.com; 2 Department of Nutritional Sciences, Rutgers University, New Brunswick, NJ 08901, USA; vquick@njaes.rutgers.edu; 3 Department of Urban-Global Public Health, Rutgers University, Newark, NJ 07102, USA; sd1081@sph.rutgers.edu; 4 Department of Health Behavior, Society and Policy, Rutgers University, Piscataway, NJ 08854, USA; wackowol@sph.rutgers.edu; 5 Department of Plant Biology, Rutgers University, New Brunswick, NJ 08901, USA; robson@sebs.rutgers.edu

**Keywords:** food waste, plate waste, school lunch, cafeteria interventions, sustainability

## Abstract

This study aimed to assess change in school-based food waste after training and implementing the Smarter Lunchrooms Movement (SLM) strategies with school food service workers. This non-controlled trial was implemented in a random sample of 15 elementary and middle schools in a Community Eligibility Program school district in the Northeast, the United States. Baseline and post-intervention food waste measurements were collected at two different time points in each school (*n* = 9258 total trays measured). Descriptive statistics, independent *t*-tests, and regression analyses were used to assess SLM strategies’ impact on changes in percent food waste. The mean number of strategies schools implemented consistently was 7.40 ± 6.97 SD, with a range of 0 to 28 consistent strategies. Independent *t*-tests revealed that at pos*t*-test, there was a significant (*p* < 0.001) percent reduction (7.0%) in total student food waste and for each food component: fruit (13.6%), vegetable (7.1%), and milk (4.3%). Overall, a training session on food waste and the SLM strategies with school-based food service workers reduced school food waste. However, the extent of the training and SLM strategies to reduce food waste varied on the basis of the consistency and type of strategies implemented.

## 1. Introduction

Food waste is a significant issue in the United States. The United States Department of Agriculture (USDA) estimates that over one-third of food produced is being wasted annually, which equates to 133 billion pounds and USD 161 billion [1,2]. In 2012, food waste contained an estimated 1217 calories and 33 g of protein per capita per day and many vital nutrients such as dietary fiber, vitamin D, and calcium [3]. In addition to the economic and nutritional cost of food waste, it also has a severe environmental cost [4]. The resources used to produce food such as land, water, and transport are not often factored into the cost of food waste [1], nor are the greenhouse gases that are released during the production, processing, transportation, and refrigeration of wasted food [1]. Thus, the negative impacts of food waste are environmental, nutritional, and economic, leading to the federal government’s focus on this issue [1,5].

In 2015, the USDA created a Food Waste Challenge jointly with the Environmental Protection Agency (EPA) to reduce food waste nationally [6]. This Food Waste Challenge aims to improve agricultural, manufacturer, and retailer practices and consumer behaviors to reduce food waste. Moreover, it has identified schools as a priority sector to reduce food waste [7,8] due to their large food service operations focusing on schools that receive funding from USDA through the National School Lunch Program [9]. The National School Lunch Program provides free and reduced meals to over 3 million children in the U.S. per day [10]. Through a new provision in the Healthy Hunger-Free Kids Act, schools with a disproportionately high percentage (i.e., 40%) of low-income students may provide free lunch to all their students through the Community Eligibility Provision (CEP) [11,12]. Schools with CEP may also be of interest when examining strategies to reduce food waste.

Prior studies have assessed the amount of plate waste with food waste ranging from 12% to 73.3% [13,14,15,16,17,18] of food components selected. Many of these studies found that vegetable waste was the highest percentage of food wasted, and higher than any other food component [14,16,19], with vegetable waste ranging from 29% to 73.3% [16,19]. However, some studies found grain waste to be the highest component wasted at 44.6% among elementary school students and fresh fruit to be the highest component wasted at 47.4% among middle school students [15]. Additionally, some studies have examined changes to the lunchroom environment, such as the Smarter Lunchrooms Movement (SLM) strategies and their effect on student consumption of more fruits, vegetables, and low-sugar milk varieties [13,17,20,21,22,23,24]. The SLM is a behavioral economics-based program that provides schools with a list of small, low- and no-cost changes they can implement in their school lunchroom to improve healthy food intake in children [25]. These SLM strategies include, for example, changes to the names of certain foods to make them more appealing to children (e.g., X-ray vision carrots or power peas) or displaying healthier food options more prominently in the lunchroom. Hence, children are more likely to purchase and consume them (e.g., providing fruit on multiple locations in the serving line) [22]. These prior studies using the SLM strategies in their multifactorial interventions have led to an increase in children’s consumption of fruits, vegetables, and low-sugar milk varieties [17,20,22,24,26,27,28]; however, findings have not been consistent across studies [26,29,30,31]. Additionally, very few school-based intervention studies have measured food waste of the entire meal (i.e., with every meal component: fruit, vegetable, milk, grain, and protein) [18,27,28] with a large and diverse number of students [14,17,32,33,34,35].

Despite many studies evaluating behavioral economic strategies to reduce plate waste among schools with high percentages of students who receive free or reduced lunch, no study to the authors’ knowledge has evaluated the use of multiple SLM strategies in a CEP school district using a comprehensive assessment. Thus, the objective of this pre-/pos*t*-test intervention was to assess the change in food waste in a low-income CEP school district located in a northeastern city in the United States after a training session on food waste with food service workers and the implementation of selected SLM strategies. Furthermore, the study aims to examine the effect of the number and types of SLM strategies implemented by food service workers on the percent reduction of school food waste. It was hypothesized that food waste would decrease in all food categories (i.e., fruit, vegetable, entrée (includes grain and protein), and milk) post-intervention, and the more consistent strategies that schools implemented, the higher the percent reduction in food waste.

## 2. Materials and Methods

### 2.1. Participants and Setting

The program was implemented and evaluated in a low-income CEP school district. The school district is located in a midsize city in the northeastern United States and serves 25,010 students from diverse backgrounds. The ethnic breakdown for the student body district-wide is 22.6% Black, 67.7% Hispanic, 4.6% White, 4.9% Asian American, 0.1% Indian/Alaskan Native, 0.1% Hawaiian Native/Pacific Islander, and 0.1% Multi-ethnic or multi-racial. Since these schools are eligible for the CEP, all students receive a free lunch, and all CEP schools were required to implement the Offer vs. Serve guidelines by the USDA.

Only elementary and middle schools that prepare their meals on-site were eligible for the current study. Of the school district’s 46 total schools, 30 met the study inclusion criteria, and 15 of the 30 eligible schools were randomly selected to participate. The chosen schools varied in total enrollment, ranging from 138 to 857 students per school with a mean of 506 students per school.

### 2.2. Intervention

The intervention included a training session in March 2017 with the food service workers and lunchroom monitors of the selected 15 schools. The training session began by introducing the issue of food waste in America and provided a snapshot of food waste observed in schools. The training included best practices for implementing low-cost or no-cost changes to the lunchroom through SLM strategies. Additionally, the training provided information on the USDA’s Offer vs. Serve guidelines, which state that each student only needs to take three of the five components (fruit, vegetable, protein, grain, and dairy) including at least one fruit or vegetable, for the meal to be reimbursed by the USDA [36]. The training provided recommendations for the food service workers to only offer the food and not serve students until they made a selection based on what they wanted. The food service workers then formed school teams, which were composed of a food service manager and two food service workers from each school, where they created a plan for implementing as many of the SLM strategies as possible in their own school [37]. This created a range of implemented strategies among schools. A total of 60 strategies were reviewed and provided to workers on a SLM scorecard during the training. Of the 60 total SLM strategies, 29 resonated with the food service staff as being able to implement in their cafeterias. These 29 strategies were under the categories: “improve the lunchroom atmosphere,” “focus on fruit,” “vary the vegetable,” and “move more milk” on the scorecard [38]. Thus, these schools were only assessed on the 29 SLM strategies that applied to food service workers.

During a two-week period following the training, the research team provided technical assistance to the staff by visiting each school to gauge how well the changes they agreed to adopt were implemented and provided support with any encountered challenges to facilitate full implementation of the school’s plan. During the post-intervention measurement of food waste (as described further below), the research staff identified the SLM strategies actually being implemented during the lunch period at each of the schools at two time periods, pos*t*-test 1 and pos*t*-test 2.

### 2.3. Measures

Baseline food waste measurements were collected at two different time points (M = 10.53 days apart) in each school the month before the intervention (from February until March of 2017). Post-intervention food waste was measured at two different time points (*M*= 15.33 days apart) in each school four weeks after the training (April 2017). The researchers felt the data were more generalizable by assessing food waste at multiple time points both before and after the intervention [39]. National school lunch programs are designed to meet students’ nutritional needs over one week; therefore, food waste for one full week is a common timeframe in school food waste studies [14,15,17,35,40]. However, due to limited resources, this study only assessed food waste twice during both the pre- and pos*t*-test periods.

### 2.4. Data Collection

Food waste weight measurements were collected by a trained research team of six interns and three food service field managers according to standard food waste measurement methods. Weight measurements are considered the gold standard in food waste measurements [41]. The team was instructed to measure each individual food item on the tray using a scale. All weights were measured using an Alegacy scale, model 74855, and measured in ounces to the nearest tenths decimal place on calibrated scales as per the manufacturer’s instructions. During training, the research team was required to practice the weighing procedure for consistency purposes. Research team members were given three practice food trays with four items on each tray to measure inter-rater reliability (IRR). Inter-rater reliability was calculated using the intraclass correlation coefficient (ICC) for absolute agreement. The IRR for the nine researchers was 1.00 for absolute agreement.

On the days of data collection, the research team obtained a sample tray from the cafeteria manager to be measured and weighed before the first lunch period. The sample tray was used to determine the average serving weight of each component. For each of the school’s data collection assessments, the research team measured and recorded approximately 10% of trays from each of the three lunch periods at random. The number of sample trays was determined before arriving at the school using the average student enrollment as a proxy for how many trays needed to be measured at each lunch period. Research staff completed a data sheet for each tray where all information (weight of food waste and type of food) was stored. For each tray, each food component—i.e., the milk, fruit, vegetable, and entrée, which consisted of a grain and protein—was measured separately. Only the edible portions of food were measured.

Additionally, given that this school district used the Offer vs. Serve guidelines if a menu item was not served to a child, the research team noted that there was no evidence (food remnants, peel, cores, etc.) of the item on the individual tray. Student personal information was not collected. After data were collected, the research team entered the data into an online survey, Qualtrics XM, which was then reviewed a second time for data entry accuracy before further analysis. The authors’ Institutional Review Board (FWA00003913) approved the study protocol (protocol number E17-416).

### 2.5. Data Analysis

Descriptive statistics were used to describe the number and types of strategies implemented post-intervention consistently by schools and the mean percent of food wasted at pre- and pos*t*-test intervention by food type (fruit, vegetable, milk, entrée). To assess percent food waste, an assessment of selection must first determine if there were significant changes in selection per food component during pre- to post-intervention. Spearman rank-order correlations and percent change of each food component selected at pre- and pos*t*-test were performed to determine whether the retraining on the USDA Offer vs. Serve guidelines might have affected the results. To calculate the percent, each food component’s weight was divided by the reference weights and multiplied by 100. Independent *t*-tests were also conducted to assess significant pre- to pos*t*-test intervention differences in the average percent waste of each food component.

Multi-linear regression analyses examining the relationship between the number of SLM strategies implemented consistently and the change in the percent of food wasted at pos*t*-test were performed. Consistently implemented strategies were observed as being implemented at pos*t*-test 1 and pos*t*-test 2. School size may influence their ability to implement SLM strategies. Additionally, schools could have some variability with attributes that affect percent food waste, such as food service staff skills and experience. For these reasons, subsequent regression analyses adjusted for school and school size (i.e., number of students enrolled) as potential confounding variables.

A series of multi-linear regression analyses were performed to assess the relationships between the total number and type of “improve the lunchroom atmosphere” SLM strategies consistently implemented and the mean percent change in total food waste. Additional multiple multivariate regression analyses were performed to separately assess the relationships of the total and type of SLM strategies consistently implemented for fruit, vegetable, and milk components with the mean percent change in total food waste for that food component at post-intervention. Since there are no specific SLM strategies for entrées, an analysis of the entrée was not included. A statistical significance level of *p* < 0.05 was used for all analyses. Data analyses were conducted in the Statistical Program for Social Sciences (IBM Corp. Released 2019. IBM SPSS Statistics for Windows, Version 26.0. Armonk, NY, USA: IBM Corp) ).

## 3. Results

The strategies most implemented by food service workers were defined as strategies where at least seven schools (46.6%) implemented the strategy at post-assessment one and two. These top strategies implemented for the food components included, “at least two kinds of fruit offered,” “milk cases/coolers are kept full throughout meal service,” and “white milk is offered in all beverage coolers” For the lunchroom atmosphere category of strategies, the top strategies implemented were, “cafeteria staff smile and greeted students upon entering the service line and throughout meal service,” “all lights in the dining and meal service areas work and are turned on,” “trash cans are at least 5 feet away from dining students”, and “trash cans are emptied when full.” There was a mean of 14.27 ± 9.42 SD strategies implemented per school at the first pos*t*-test. About two weeks later, at the second pos*t*-test, the mean number of strategies implemented per school was 11.00 ± 6.96 SD. The mean number of strategies schools implemented consistently across both pos*t*-tests was 7.40 ± 6.97 SD, with a range of 0 to 28 consistent strategies.

Selection of food components at pre- and post-intervention is needed to assess the impact of the changes in percent food wasted. The total number and the percentage of selected items before and after the intervention for each component were calculated. As shown in Table 1, more fruit, vegetable, and entrée components were selected at pos*t*-test, while milk selection decreased slightly. The only significant correlation between pre- and pos*t*-test food components selected were vegetables (*r* = −0.20, *p* > 0.001) and fruits (r = −0.02, *p* < 0.05); however, these correlations were relatively weak (*r* < 0.30).

This section is divided by subheadings. It should provide a concise and precise description of the experimental results, their interpretation, as well as the experimental conclusions that can be drawn.

A total of 9258 trays were measured for food waste: 4642 trays at the pre-test and 4616 trays at the pos*t*-test intervention. There were reductions in the average percent of food wasted per tray (Table 1). Independent *t*-tests revealed a significant percent reduction in all food components (*p* < 0.005) at pos*t*-test (Table 2).

Multiple linear regression assessing changes in percentage of total food waste pre- and post-intervention while controlling for school effects and total enrollment found that food waste significantly decreased by 7.01% (β = −7.061, *p* < 0.001) with the school effect being significantly different by 0.25% (β = 0.247, *p* < 0.01). Similarly, percent fruit waste pre- and post-intervention decreased by 13.6% (β = −13.631, *p* < 0.001) with effects for school (β = 0.308, *p* < 0.05) and total enrollment (β = 0.012, *p* < 0.001). Vegetable waste also decreased significantly post-intervention with a 7.11% (β = −7.112, *p* < 0.001) decrease after controlling for school effects and total enrollment. Milk waste decreased by 4.3% (β = −4.33, *p* < 0.001) with effects for school (β = 0.285, *p* < 0.01) but not total enrollment. Finally, entrée waste significantly decreased by 2.1% (β = −2.146, *p* < 0.01) with significant effects for total enrollment (β = −0.011, *p* < 0.001) and school effect (β = 0.471, *p* < 0.001).

### Effect of SLM Strategies on Food Waste

In the multiple linear regression analysis, there was a significant association between the number of total consistent strategies implemented and percent changes in total food waste (β = −0.416, *p* < 0.001), when including school (β = 0.385, *p* < 0.001) and total enrollment (β = 0.002, *p* = 0.452) as confounding variables. For every additional strategy that a school implemented consistently, the percent of total food waste decreased by 0.42%. School effects and total enrollment were significant in most regression models; however, the magnitude of the association was always less than 1%. Therefore, it was not reported in subsequent regression models.

Separate multiple and multivariate linear regression analyses were conducted to assess the influence of the “improve the lunchroom atmosphere” SLM strategies on percent total food waste (Table 3). The total number of lunchroom environment strategies consistently implemented was negatively associated with the percent total food waste (β = −1.132, *p* < 0.001). Overall, 7 of the 10 individual “improve the lunchroom atmosphere” SLM strategies (i.e., posting a menu board with tomorrow’s featured meal) were found to be significantly associated with decreased percent total food waste, as seen in Table 3. Three of the seven strategies were found to significantly (*p* < 0.05) increase food waste (range of 6.38% to 9.26% increase). However, the remaining four strategies significantly (*p* < 0.001) decreased the percent of total food waste (with decreases ranging from 2.63% to 17.48%).

Table 4 shows the associations between food component-specific strategies and food component-specific waste reduction. With every additional fruit SLM strategy implemented consistently, a 0.76% increase in total fruit waste was observed (β = 0.755, *p* = 0.030). Additionally, the total number of vegetable strategies consistently implemented significantly decreased total vegetable waste by 2.60% (β = −2.569, *p* < 0.001). Finally, the consistent implementation of total milk strategies significantly decreased milk waste by 1.17%. In multivariate regression analyses, there were significant associations of four fruit-specific strategies with total fruit waste, with two strategies significantly (*p* ≤ 0.001) increasing waste and two strategies significantly (*p* ≤ 0.001) decreasing waste. Similarly, there were significant associations of six specific vegetable strategies with total vegetable waste in multivariate regression analyses, but only three of these strategies significantly (*p* < 0.001) decreased total vegetable waste. Lastly, in multivariate regression analyses, there were significant associations of three specific milk strategies with total milk waste, but only one of these milk strategies significantly (*p* < 0.001) decreased total milk waste.

## 4. Discussion

This study aimed to assess food waste change in a low-income CEP school district after a training session on food waste with food service workers and the SLM strategies’ implementation. To assess differences in waste, it is essential first to consider differences in selection. The pre- and post-assessment indicated an increase in fruits and vegetables selection by 1.6% and 15.6%, respectively, indicating that with improved quality and fresh options available, more students selected fruits and vegetables at pos*t*-test. As evidenced in the results, the food service training program was associated with reduced total student plate waste, i.e., fruit, vegetable, entrée, and milk percent waste, by 7.0%. Additionally, for every SLM strategy implemented, total student food waste decreased by 0.4%. Reducing student food waste in these food categories can be significant since many studies indicate that fruits, vegetables, and milk are wasted at high percentages (22% to 46.8% for fruit, 29% to 73.3% for vegetables, and 16% to 45.5% for milk) [7,13,15,16,19,42,43] while also being essential contributors to key nutrients [3]. In this study, an increase in selection of fruits and vegetables as well as a decrease in fruit and vegetable waste indicates that the students consumed more fruits and vegetables after the intervention. Even though this was not the central focus of this study, it is an important finding to consider.

Additionally, there were reductions in student food waste after the intervention, with a 7.0% reduction in total waste, 13.6% reduction in fruit waste, 7.1% reduction in vegetable waste, and 4.3% reduction in milk waste. This was similar to a prior study that assessed the Smarter Lunchroom initiative’s impact on food waste [17]. Their team found a 19.5% reduction in total student food waste, a 9.4% reduction in fruit waste, and a 9.0% reduction in vegetable waste [17]. Their change in percent of food waste was not significant, which may be due to the small sample size (*n* = 43) [17]. Whereas in this study, a significant change was detected in food waste with a large sample size. This may indicate that the SLM effect on food waste is small and can only be detected only in large sample sizes.

It was hypothesized that schools with food service workers implementing more strategies consistently would have a higher reduction in food waste. The total number of strategies consistently implemented was found to significantly reduce percent food waste by only 0.42%, even though most schools, on average, consistently implemented 7 of the 29 strategies targeting food service staff. These findings suggest that food service workers can reduce food waste by a small amount with minimal strategies if implemented consistently. Thus, giving food service workers the autonomy to choose their SLM strategies and providing a bottom-up approach towards making environmental changes in the cafeteria may have influenced the significant decrease in food waste.

It was also hypothesized that implementing food-specific strategies would reduce waste for that specific food. For fruit SLM strategies, increasing the total number of consistent fruit strategies implemented was significantly associated with a slight increase in percent fruit waste of 0.76%. However, when assessing specific fruit SLM strategies with total fruit waste, it is evident that strategies such as offering fruit in multiple locations or identifying the fruit of the day can significantly decrease the percent fruit waste by as much as 8.6%. Meanwhile, other strategies, such as offering multiple kinds of fruit or offering sliced or cut fruit, significantly increased the percent fruit waste by as much as 9.5%. These findings were unexpected as previous studies that have assessed the increase in the number of fruits offered [20,44] and slicing fruit [20] strategies have found that students typically consumed more fruit at post-intervention: 27.0% more in one study [44]. These differences may be due to the types and quality of fruits offered, as canned fruit was considered sliced or cut fruit in the current study. However, the strategies that reduced fruit waste in this study, such as changing the location of where fruits are offered, are supported by a previous study that assessed the impact of providing fruit in a different location to improve children’s fruit consumption [27]. In this study, Adams and colleagues found that, by placing fruit directly on the serving line, students consumed 4.8 times more fruit more than the control group [27]. However, findings from this study suggest that changing the location of fruit can also decrease fruit waste in addition to increasing consumption.

Conflicting strategy results were found for milk and vegetable SLM strategies. Despite the total number of consistent vegetable and milk strategies significantly decreasing vegetable and milk waste, individual strategy effects conflicted, with some strategies increasing waste while others decreased waste. This is surprising given the body of evidence supporting increased vegetable consumption when implementing various vegetable SLM strategies [17,20,45,46,47] These conflicting results may be due to the small number of schools implementing some of these strategies in this study sample. Only one or two schools implemented the vegetable strategies that led to increased total vegetable food waste. Thus, this may reduce the generalizability of these results. However, the milk findings were not surprising, as there is no evidence from prior work that the SLM strategies affect reducing milk waste and conflicting results on how SLM strategies effect milk consumption.

Additionally, studies on the effect of SLM strategies on the selection or consumption of milk have varied considerably [7,24,29,48,49]. Most studies have found that the SLM strategies increased milk selection or sales [29,48,50]; however, limited evidence suggests SLM strategies increase milk consumption [20,29]. One recent study [24] found that sugar-sweetened milk consumption decreased by 10% in the treatment group.

## 5. Conclusions

### 5.1. Limitations

Overall, it may be difficult to assess the effect of individual aspects of the training on food waste due to the complexity of evaluating packages of interventions such as SLM strategies. However, perhaps there is a synergistic effect of this training that reinforces the strategies to collectively reduce food waste. Even though significant associations of fruit or vegetable SLM strategies were found in this study inconsistently, when combined with other strategies and consistently implemented across two-time points, which may have contributed to the overall effect on decreased food waste. Due to the small sample of schools in this study, this relationship could not be explored further. Additionally, due to the nature of the training, food service staff could choose the strategies they wanted to implement; therefore, some strategies were implemented in multiple schools while others were only implemented in one or two schools.

Moreover, the definition of consistent use of a strategy in this study was defined by whether food service staff implemented the strategy on two occasions over weeks. However, accurate consistency measures assessed over multiple occasions and months should be considered in future work. Moreover, the use of ounces instead of the use of grams can be a limitation in the food waste audits as ounces may not be as precise.

Additionally, the wording of some of the SLM strategies may have made it difficult for researchers to assess accurately. Many of the SLM scorecard strategies contained multiple criteria—e.g., “A variety of fruit was offered in an attractive bowl or basket.” If a lunchroom contained a variety of fruit but not in attractive bowls, this strategy would not be considered implemented. Similarly, if the lunchroom offered a single fruit in attractive bowls or baskets, it would not be considered an implemented strategy. Therefore, revising the SLM strategies to remove multiple criteria statements could help improve the assessment’s accuracy.

Despite being trained on the Offer vs. Serve guidelines, the selection of fruits, vegetables, and the entrée increased following the intervention implementation. It was anticipated that the decrease in vegetable and milk waste was due to decreased selection by students rather than the SLM strategies’ effects. That does not seem to be the case, as fruit and vegetable selection increased after the training.

Although this study did not use an experimental design, it was strengthened by using multiple assessments before and after the intervention to demonstrate temporal sequencing of events and minimize any bias potentially introduced by data collected from a single day or meal offering. Despite the menus not matching completely in the pre- and post-assessment measures, they were matched as much as possible between the 15 schools. Additionally, students, staff, and principals who consented to the study were not given notice of when the research team would be coming for visits. All the measurements happened within the same year to reduce the threat of maturation. Although this study was limited to 15 schools in a single district, this study’s major strength is the large sample size of trays assessed and rigorous weight methods used for assessing food waste and controlling for school and enrollment effects in the analyses.

### 5.2. Implications for Research and Practice

The findings demonstrated that a training session on food waste with the engagement of food service workers and the SLM strategy implementation led to a reduction in food waste in a low-income CEP school district. However, the extent to which Smarter Lunchroom Movement strategies were consistently implemented over two post-training visits in schools varied. More research on the implementation of food waste reduction strategies may help explain some of the study findings and improve the implementation of this program for other school districts to utilize in the efforts to reduce school food waste. Additionally, studies with larger sample sizes are needed to assess the impact of individual strategies further when implemented together, as there may be synergistic effects. Moreover, a study designed for causal inferences is needed for more conclusive data. Finally, an assessment of a top-down approach, i.e., management requiring food service staff to implement SLM strategies rather than a bottom-up approach, where food service workers are empowered to make the changes, may help understand why only a few strategies implemented consistently may have had a significant decrease in food waste overall.

## Figures and Tables

**Table 1:** Total Number of Items Selected by Food Component at Pre- and Pos*t*-test Intervention (*n* = 9258).

Food Item	Pre-Test Items Selected ^a^ n (% of Total Trays Measured in Which the Item Was Selected)	Pos*t*-test Items Selected ^b^ n (% of Total Trays Measured in Which the Item Was Selected)	Percentage Point Difference	Spearman Correlations ^c^
Fruits	2686 (57.9%)	2748 (59.5%)	1.6%	−0.017
Vegetables	3468(74.7%)	4166 (90.3%)	15.6%	−0.204 ***
Milk	3374 (72.7%)	3325 (72.0%)	−0.7%	0.007
Entrée (Protein and Grain)	4582 (98.7%)	4570 (99.0%)	0.7%	−0.014
Total Number of Trays	4642	4616		

^a^ Summed selected food items measured by evidence that the food item was present on tray for day 1 and day 2 pre-intervention; ^b^ summed selected food items measured by evidence that the food item was present on tray for day 3 and day 4 post-intervention; ^c^ Spearman rank-order correlations examining correlations of items selected between pre- and post-intervention. *** *p* < 0.001.

**Table 2:** Differences in Average Percent of Food Waste by Food Component per Tray at Pre- and Pos*t*-test Intervention (*n* = 9258).

		Pre-Test		Pos*t*-test				
Food Item	% Measured Weight ^a^ Day 1	% Measured Weight ^a^ Day 2	% Average Measured Weight ^a^	% Measured Weight ^a^ Day 1	% Measured Weight ^a^ Day 2	% Average Measured Weight ^a^	Independent *t*-Tests ^b^	
	Mean ± SD ^c^	Mean ± SD ^c^	Mean ± SD ^c^	Mean ± SD ^c^	Mean ± SD ^c^	Mean ± SD ^c^	*t*	*p*-Value
Fruits (*n* = 5434)	8.14 ± 39.27	55.83 ± 44.03	**1.61 ± 42.30**	46.17 ± 44.47	49.54 ± 44.84	**47.89 ± 44.69**	−11.62	<0.001
Vegetables (*n* = 7634)	74.21 ± 36.18	70.58 ± 38.94	**2.39 ± 37.63**	60.68 ± 41.54	69.48 ± 39.54	**65.17 ± 40.77**	−7.98	<0.001
Milk (*n* = 6699)	49.41 ± 40.58	34.94 ± 40.36	**46.37 ± 40.55**	42.36 ± 41.09	41.45 ± 40.65	**41.89 ± 40.86**	−4.50	<0.001
Entrée (*n* = 9152) (Protein + Grain)	29.46 ± 34.97	37.72 ± 38.46	**33.69 ± 37.03**	32.75 ± 38.12	30.08 ± 35.31	**31.40 ± 36.76**	−2.97	<0.005

^a^ Standard food waste measurement method. ^b^ Independent *t*-tests examining significant differences between pre- and pos*t*-test intervention of the percent average measured tray weights of each food component. ^c^ mean and standard deviation calculation is based on percent of food items that were selected.

**Table 3:** Coefficient Estimates for Multiple Linear and Multivariate Regression Analyses Examining Associations between the Total Number and Type of Consistent Lunchroom Environment Strategies Implemented with Percent Change in Total Food Waste (*n* = 15).

	Percent Change in Total Food Waste (%)	
Independent Variables ^a^	Β ^b^	*p*-Value
Total Number of Consistent Lunchroom Environment Strategies Observed in Both Post-Training Visits.	−1.13	<0.001 ***
Lunchroom Environment Strategy 1: “Cafeteria staff smile and greet students upon entering the service line and throughout meal service” (*n* = 7)	−2.63	<0.001 ***
Lunchroom Environment Strategy 2: “Attractive, healthful food posters are displayed in dining and service areas” (*n* = 4)	−17.49	<0.001 ***
Lunchroom Environment Strategy 3: “A menu board with today’s featured meal options with creative names is readable from 5 ft away when approaching the service are” (*n* = 3)	6.38	0.019 *
Lunchroom Environment Strategy 4: “The lunchroom is branded and decorated in a way that reflects the student body” (*n* = 2)	−14.41	<0.001 ***
Lunchroom Environment Strategy 5: “Cleaning supplies or broken/unused equipment are not visible during meal service” (*n* = 5)	−12.24	<0.001 ***
Lunchroom Environment Strategy 6: “All lights in the dining and meal service areas work and are turned on” (*n* = 7)	9.26	<0.001 ***
Lunchroom Environment Strategy 7: “Compost/recycling and trash cans are at least 5 feet away from dining students” (*n* = 4)	2.94	0.292
Lunchroom Environment Strategy 8: “There is a clear traffic pattern. Signs, floor decals, or rope lines are used when appropriate” (*n*= 3)	n/a	n/a
Lunchroom Environment Strategy 9: “Trash cans are emptied when full” (*n* = 7)	6.52	<0.001 ***
Lunchroom Environment Strategy 10: “A menu board with tomorrows featured meal with creative names is readable from 5 ft away in the service or dining area” (*n* = 1)	−0.74	0.854

^a^ Multiple linear regression analyses were run separately for consistent total number of strategies, and a multivariate regression was used for the strategy type variables while controlling for school and enrollment effects. ^b^ Unstandardized beta coefficient. * *p* < 0.05, *** *p* < 0.001; n/a: variable was excluded from the multiple linear regression analysis.

**Table 4:** Coefficient Estimates for Multiple Linear and Multivariate Regression Analyses Examining Associations Between the Total Number and Type of Consistent Strategy Implemented with Percent Change in Total Food Waste per Food Component (*n* = 15).

	Percent Change in Total Food Waste Per Food Component (%)	
Independent Variables ^a^	β ^b^	*p*-Value
Total Number of Consistent Fruit Strategies	0.76	0.030 *
Fruit Strategy 1: “At least two fruits were offered” (*n* = 9)	7.61	0.001 **
Fruit Strategy 2: “Sliced or cut fruit was offered” (*n* = 4)	9.46	*p* < 0.001 ***
Fruit Strategy 3: “A variety of fruit was offered in an attractive bowl or basket” (*n* = 6)	0.24	0.914
Fruit Strategy 4: “Fruit was offered in at least two locations” (*n* = 3)	−8.63	*p* < 0.001 ***
Fruit Strategy 5: “At least one fruit was identified as the fruit of the day” (*n* = 1)	n/a	n/a
Fruit Strategy 6: “A fruit taste test was offered” (*n* = 1)	−8.37	0.001 **
Total Number of Consistent Vegetable Strategies	−2.60	*p* < 0.001 ***
Vegetable Strategy 1: “At least two kinds of vegetables are offered” (*n* = 1)	n/a	n/a
Vegetable Strategy 2: “Vegetables are offered on all service line” (*n* = 4)	25.55	*p* < 0.001 ***
Vegetable Strategy 3: “Both hot and cold vegetables are offered” (*n* = 3)	−23.39	*p* < 0.001 ***
Vegetable Strategy 4: “When cut, raw vegetables are offered, they are paired with a low-fat dip such as ranch, hummus, or salsa” (*n* = 3)	−43.30	*p* < 0.001 ***
Vegetable Strategy 5: “A serving of vegetables is incorporated into an entrée item at least once a month” (*n* = 2)	−18.45	*p* < 0.001 ***
Vegetable Strategy 6: “Self-serve spices and seasonings are available for students to add flavor to vegetables” (*n* = 1)	n/a	n/a
Vegetable Strategy 7: “At least one vegetable is identified as the featured vegetable-of-the-day and is labeled with a creative, descriptive name at the point of selection” (*n* = 2)	21.56	*p* < 0.001 ***
Vegetable Strategy 8: “A vegetable taste test is offered at least once a year” (*n* = 1)	26.02	*p* < 0.001 ***
Total Number of Consistent Milk Strategies	−1.17	0.001 **
Milk Strategy 1: “Milk cases/coolers are kept full throughout meal service” (*n* = 8)	4.90	*p* < 0.001 ***
Milk Strategy 2: “White milk is offered in all beverage coolers” (*n* = 9)	0.12	0.944
Milk Strategy 3: “White milk is organized and represents at least 1/3 of all milk in each designated milk cooler” (*n* = 5)	−14.29	*p* < 0.001 ***
Milk Strategy 4: “White milk is displayed in front of other beverages in all coolers” (*n* = 5)	4.46	0.001 **
Milk Strategy 5: “1% or non-fat white milk is identified as the featured milk and is labeled with a creative, descriptive name” (*n* = 0)	n/a	n/a
n/a: variable was excluded from the multiple multivariate regression analysis		

^a^ Multiple multivariate regression analyses were run separately for consistent total number of strategies, and a multivariate regression was run for strategy types per food component while controlling for school and enrollment effects. ^b^ Unstandardized beta coefficient. * *p* < 0.05, ** *p* < 0.01, *** *p* < 0.001.

## Data Availability

Please email primary author for dataset.
